# Supplementary material for: EnzML: multi-label prediction of enzyme classes using InterPro signatures
Source: BMC Bioinformatics. 2012 Apr 25;13:61. doi: 10.1186/1471-2105-13-61 (PMC3483700; doi:10.1186/1471-2105-13-61)
Supplement: Addtional file 5 — The Java code to format the data files, evaluate and predict. The file enzml_java_code.tar.gz contains the Java code used to format database data to ARFF and XML formats, to execute cross and train-test (jackknife) evaluations and to record evaluation results to database. More information is included in the readme.txt file and the Javadoc files. The code can be used with a MySQL database. To use a different database software, other JDBC drivers might be required. [file 1471-2105-13-61-S5.gz › java_code/enzml2011/doc/test/mulan/MulanArffTest.html]

MulanArffTest


---


|  |  |  |
| --- | --- | --- |
| **Overview** \| **Package** \| **Class** \| **Use** \| **Tree** \| **Deprecated** \| **Index** \| **Help** | |  |
| **PREV CLASS**   **NEXT CLASS** | **FRAMES**    **NO FRAMES**     **All Classes** |  |
| SUMMARY: NESTED \| FIELD \| CONSTR \| METHOD | DETAIL: FIELD \| CONSTR \| METHOD |  |


---


## test.mulan Class MulanArffTest

```
java.lang.Object
  junit.framework.Assert
      junit.framework.TestCase
          test.mulan.MulanArffTest
```

**All Implemented Interfaces:**: junit.framework.Test

---

``` public class MulanArffTest extends junit.framework.TestCase ```

Class

**Version:**
:   24 Feb 2011

**Author:**
:   Luna De Ferrari luna.deferrari-at-ed.ac.uk

---

| **Field Summary** | |
| --- | --- |
| `static java.lang.String` | `arff_EC_Instance1` |
| `static java.lang.String` | `arffInstance1` |
| `static java.lang.String` | `arffInstance2` |
| `static java.lang.String` | `arffInstance4` |
| `static java.lang.String` | `arffInstanceAttributeVoid` |
| `static java.lang.String` | `arffInstanceClassAttributeVoid` |
| `static java.lang.String` | `arffInstanceClassVoid` |
| `static java.lang.String` | `ATT_EC_1_TO_STRING` |
| `static java.lang.String` | `ATT_EC_11_TO_STRING` |
| `static java.lang.String` | `ATT_EC_111_TO_STRING` |
| `static java.lang.String` | `ATT_EC_1111_TO_STRING` |
| `static java.lang.String` | `ATT_EC_2_TO_STRING` |
| `static java.lang.String` | `ATT_EC_22_TO_STRING` |
| `static java.lang.String` | `ATT_EC_222_TO_STRING` |
| `static java.lang.String` | `ATT_EC_2222_TO_STRING` |
| `static java.lang.String` | `ATT1_TO_STRING` |
| `static java.lang.String` | `ATT2_TO_STRING` |
| `static java.lang.String` | `ATT3_TO_STRING` |
| `static java.lang.String` | `ATT4_TO_STRING` |
| `static java.lang.String` | `ATT5_TO_STRING` |


**Constructor Summary**

`MulanArffTest()`


| **Method Summary** | |
| --- | --- |
| `static int` | `generateTestArffAndReturnId(java.lang.String arffPropsPath)`             Create test arff file |
| `static java.lang.String` | `getExpectedMulanTestFile()` |
| `static java.lang.String` | `getExpectedMulanTrainFile()` |
| `static java.lang.String` | `getExpectedMulanXml2()` |
| `static java.lang.String` | `getExpectedMulanXmlTestFile()` |
| `static java.lang.String` | `getExpectedMulanXmlTrainFile()` |
| `static int` | `getFirstTestArffFromDb()`             public static int getFirstFilteredTestArff() { ArffPropsTableReader reader = ArffPropsTableReaderTest.getReader1(); // return the first arff having different identifiers as "old test id" // and "id the attributes were taken from (train set)" // return the first arff id containing "test" in its pathname return reader.getIdByArffPath("\_with\_attr\_of\_"); } public static int getFirstFilteredTrainArff() { ArffPropsTableReader reader = ArffPropsTableReaderTest.getReader1(); // return the first arff having different identifiers as "old test id" // and "id the attributes were taken from (train set)" // return the first arff id containing "test" in its pathname return reader.getIdByArffPath("\_with\_attr\_of\_"); } |
| `static int` | `getFirstTrainArffFromDb()` |
| `static int` | `getFirstValidArff2FromDb()` |
| `static MulanArff` | `getLoadedMulan(java.lang.String arffPropsPath)` |
| `static MulanArff` | `getMulan(java.lang.String arffPropsPath)` |
| `static MulanArff` | `getMulanArff1()` |
| `static MulanArff` | `getMulanArff1LoadedAndWritten()` |
| `static MulanArff` | `getMulanArffTest()` |
| `static MulanArff` | `getMulanArffTestLoadedAndWritten()` |
| `static MulanArff` | `getMulanArffTrain()` |
| `static MulanArff` | `getMulanArffTrainLoadedAndWritten()` |
| `static MulanArff` | `getWrittenMulan(java.lang.String arffPropsPath)` |
| `void` | `testGenerateArffFileFromDb2()`             public static String getExpectedMulanArff1() { return "@relation test\_data\n\n" + MulanArffTest.ATT1\_TO\_STRING + "\n" + MulanArffTest.ATT2\_TO\_STRING + "\n" + MulanArffTest.ATT3\_TO\_STRING + "\n" + MulanArffTest.ATT4\_TO\_STRING + "\n" + MulanArffTest.ATT5\_TO\_STRING + "\n" + "@attribute class1 {0,1}\n" + "@attribute class2 {0,1}\n" + "@attribute class3 {0,1}\n" + "@attribute class4 {0,1}\n" + "\n@data\n" + "% " + DataOne.INST1 + "\n" + MulanArffTest.arffInstance1 + "\n" + "% " + DataOne.INST2 + "\n" + MulanArffTest.arffInstance2 + "\n" + "% " + DataOne.INST4 + "\n" + MulanArffTest.arffInstance4 + "\n" + "% " + DataOne.INSTATTVOID + "\n" + MulanArffTest.arffInstanceAttributeVoid + "\n" + "% " + DataOne.INSTCLASSATTVOID + "\n" + MulanArffTest.arffInstanceClassAttributeVoid + "\n" + "% " + DataOne.INSTCLASSVOID + "\n" + MulanArffTest.arffInstanceClassVoid; } public static String getExpectedMulanArff2() { String attributes = "@relation test\_ec\_data\n\n@attribute 1.-.-.- {0,1}\n@attribute 1.1.-.- {0,1}\n@attribute 1.1.1.- {0,1}\n@attribute 1.1.1.1 {0,1}\n@attribute 2.-.-.- {0,1}\n@attribute 2.2.-.- {0,1}\n@attribute 2.2.2.- {0,1}\n@attribute 2.2.2.2 {0,1}\n@attribute 3.-.-.- {0,1}\n@attribute 3.3.-.- {0,1}\n@attribute 3.3.3.- {0,1}\n@attribute 3.3.3.3 {0,1}\n@attribute att1 {0,1}\n@attribute att2 {0,1}\n@attribute att3 {0,1}\n@attribute att4 {0,1}\n@attribute att5 {0,1}\n\n"; String instances = "@data\n% inst1\n{0 1,1 1,2 1,3 1,12 1}\n% inst10\n{4 1,5 1,6 1,7 1,16 1}\n% inst11\n{4 1,5 1,6 1,7 1}\n% inst12\n{12 1}\n% inst13\n{}\n% inst2\n{4 1,5 1,6 1,7 1,14 1}\n% inst3\n{0 1,1 1,2 1,3 1,12 1}\n% inst4\n{4 1,5 1,6 1,7 1,14 1}\n% inst5\n{0 1,1 1,2 1,3 1,12 1}\n% inst6\n{4 1,5 1,6 1,7 1,14 1}\n% inst7\n{0 1,1 1,2 1,3 1,12 1}\n% inst8\n{4 1,5 1,6 1,7 1,14 1}\n% inst9\n{0 1,1 1,2 1,3 1,15 1}\n% instA\n{8 1,9 1,10 1,11 1,13 1}\n% instB\n{4 1,5 1,6 1,7 1,14 1}\n% instC\n{4 1,5 1,6 1,7 1,13 1,14 1}\n% inst\_att\_void\n{4 1,5 1,6 1,7 1}\n% inst\_class\_att\_void\n{}\n% inst\_class\_void\n{13 1}"; return attributes + instances; } |

**Methods inherited from class junit.framework.TestCase**

`countTestCases, getName, run, run, runBare, setName, toString`

**Methods inherited from class junit.framework.Assert**

`assertEquals, assertEquals, assertEquals, assertEquals, assertEquals, assertEquals, assertEquals, assertEquals, assertEquals, assertEquals, assertEquals, assertEquals, assertEquals, assertEquals, assertEquals, assertEquals, assertEquals, assertEquals, assertEquals, assertEquals, assertFalse, assertFalse, assertNotNull, assertNotNull, assertNotSame, assertNotSame, assertNull, assertNull, assertSame, assertSame, assertTrue, assertTrue, fail, fail, failNotEquals, failNotSame, failSame, format`

**Methods inherited from class java.lang.Object**

`equals, getClass, hashCode, notify, notifyAll, wait, wait, wait`

**Field Detail**

### arffInstance1

```
public static final java.lang.String arffInstance1
```

**See Also:**: Constant Field Values

---


### arff\_EC\_Instance1

```
public static final java.lang.String arff_EC_Instance1
```

**See Also:**: Constant Field Values

---


### arffInstance2

```
public static final java.lang.String arffInstance2
```

**See Also:**: Constant Field Values

---


### arffInstance4

```
public static final java.lang.String arffInstance4
```

**See Also:**: Constant Field Values

---


### arffInstanceClassAttributeVoid

```
public static final java.lang.String arffInstanceClassAttributeVoid
```

**See Also:**: Constant Field Values

---


### arffInstanceClassVoid

```
public static final java.lang.String arffInstanceClassVoid
```

**See Also:**: Constant Field Values

---


### arffInstanceAttributeVoid

```
public static final java.lang.String arffInstanceAttributeVoid
```

**See Also:**: Constant Field Values

---


### ATT1\_TO\_STRING

```
public static final java.lang.String ATT1_TO_STRING
```

**See Also:**: Constant Field Values

---


### ATT2\_TO\_STRING

```
public static final java.lang.String ATT2_TO_STRING
```

**See Also:**: Constant Field Values

---


### ATT3\_TO\_STRING

```
public static final java.lang.String ATT3_TO_STRING
```

**See Also:**: Constant Field Values

---


### ATT4\_TO\_STRING

```
public static final java.lang.String ATT4_TO_STRING
```

**See Also:**: Constant Field Values

---


### ATT5\_TO\_STRING

```
public static final java.lang.String ATT5_TO_STRING
```

**See Also:**: Constant Field Values

---


### ATT\_EC\_1\_TO\_STRING

```
public static final java.lang.String ATT_EC_1_TO_STRING
```

**See Also:**: Constant Field Values

---


### ATT\_EC\_11\_TO\_STRING

```
public static final java.lang.String ATT_EC_11_TO_STRING
```

**See Also:**: Constant Field Values

---


### ATT\_EC\_111\_TO\_STRING

```
public static final java.lang.String ATT_EC_111_TO_STRING
```

**See Also:**: Constant Field Values

---


### ATT\_EC\_1111\_TO\_STRING

```
public static final java.lang.String ATT_EC_1111_TO_STRING
```

**See Also:**: Constant Field Values

---


### ATT\_EC\_2\_TO\_STRING

```
public static final java.lang.String ATT_EC_2_TO_STRING
```

**See Also:**: Constant Field Values

---


### ATT\_EC\_22\_TO\_STRING

```
public static final java.lang.String ATT_EC_22_TO_STRING
```

**See Also:**: Constant Field Values

---


### ATT\_EC\_222\_TO\_STRING

```
public static final java.lang.String ATT_EC_222_TO_STRING
```

**See Also:**: Constant Field Values

---


### ATT\_EC\_2222\_TO\_STRING

```
public static final java.lang.String ATT_EC_2222_TO_STRING
```

**See Also:**: Constant Field Values


**Constructor Detail**

### MulanArffTest

```
public MulanArffTest()
```


**Method Detail**

### testGenerateArffFileFromDb2

```
public void testGenerateArffFileFromDb2()
```

:   public static String getExpectedMulanArff1() {
    return "@relation test\_data\n\n" + MulanArffTest.ATT1\_TO\_STRING + "\n"
    + MulanArffTest.ATT2\_TO\_STRING + "\n"
    + MulanArffTest.ATT3\_TO\_STRING + "\n"
    + MulanArffTest.ATT4\_TO\_STRING + "\n"
    + MulanArffTest.ATT5\_TO\_STRING + "\n"
    + "@attribute class1 {0,1}\n" + "@attribute class2 {0,1}\n"
    + "@attribute class3 {0,1}\n" + "@attribute class4 {0,1}\n"
    + "\n@data\n" + "% " + DataOne.INST1 + "\n"
    + MulanArffTest.arffInstance1 + "\n" + "% " + DataOne.INST2
    + "\n" + MulanArffTest.arffInstance2 + "\n" + "% "
    + DataOne.INST4 + "\n" + MulanArffTest.arffInstance4 + "\n"
    + "% " + DataOne.INSTATTVOID + "\n"
    + MulanArffTest.arffInstanceAttributeVoid + "\n" + "% "
    + DataOne.INSTCLASSATTVOID + "\n"
    + MulanArffTest.arffInstanceClassAttributeVoid + "\n" + "% "
    + DataOne.INSTCLASSVOID + "\n"
    + MulanArffTest.arffInstanceClassVoid;
    }
    public static String getExpectedMulanArff2() {
    String attributes = "@relation test\_ec\_data\n\n@attribute 1.-.-.- {0,1}\n@attribute 1.1.-.- {0,1}\n@attribute 1.1.1.- {0,1}\n@attribute 1.1.1.1 {0,1}\n@attribute 2.-.-.- {0,1}\n@attribute 2.2.-.- {0,1}\n@attribute 2.2.2.- {0,1}\n@attribute 2.2.2.2 {0,1}\n@attribute 3.-.-.- {0,1}\n@attribute 3.3.-.- {0,1}\n@attribute 3.3.3.- {0,1}\n@attribute 3.3.3.3 {0,1}\n@attribute att1 {0,1}\n@attribute att2 {0,1}\n@attribute att3 {0,1}\n@attribute att4 {0,1}\n@attribute att5 {0,1}\n\n";
    String instances = "@data\n% inst1\n{0 1,1 1,2 1,3 1,12 1}\n% inst10\n{4 1,5 1,6 1,7 1,16 1}\n% inst11\n{4 1,5 1,6 1,7 1}\n% inst12\n{12 1}\n% inst13\n{}\n% inst2\n{4 1,5 1,6 1,7 1,14 1}\n% inst3\n{0 1,1 1,2 1,3 1,12 1}\n% inst4\n{4 1,5 1,6 1,7 1,14 1}\n% inst5\n{0 1,1 1,2 1,3 1,12 1}\n% inst6\n{4 1,5 1,6 1,7 1,14 1}\n% inst7\n{0 1,1 1,2 1,3 1,12 1}\n% inst8\n{4 1,5 1,6 1,7 1,14 1}\n% inst9\n{0 1,1 1,2 1,3 1,15 1}\n% instA\n{8 1,9 1,10 1,11 1,13 1}\n% instB\n{4 1,5 1,6 1,7 1,14 1}\n% instC\n{4 1,5 1,6 1,7 1,13 1,14 1}\n% inst\_att\_void\n{4 1,5 1,6 1,7 1}\n% inst\_class\_att\_void\n{}\n% inst\_class\_void\n{13 1}";
    return attributes + instances;
    }

---


### generateTestArffAndReturnId

```
public static int generateTestArffAndReturnId(java.lang.String arffPropsPath)
```

:   Create test arff file

    :   **Returns:**: the identifier of the arff file record in the database

---


### getExpectedMulanTestFile

```
public static java.lang.String getExpectedMulanTestFile()
```

---


### getExpectedMulanTrainFile

```
public static java.lang.String getExpectedMulanTrainFile()
```

---


### getExpectedMulanXml2

```
public static java.lang.String getExpectedMulanXml2()
```

---


### getExpectedMulanXmlTestFile

```
public static java.lang.String getExpectedMulanXmlTestFile()
```

---


### getExpectedMulanXmlTrainFile

```
public static java.lang.String getExpectedMulanXmlTrainFile()
```

---


### getFirstTestArffFromDb

```
public static int getFirstTestArffFromDb()
```

:   public static int getFirstFilteredTestArff() { ArffPropsTableReader
    reader = ArffPropsTableReaderTest.getReader1(); // return the first arff
    having different identifiers as "old test id" // and
    "id the attributes were taken from (train set)"
    // return the first arff id containing "test" in its pathname return
    reader.getIdByArffPath("\_with\_attr\_of\_"); }
    public static int getFirstFilteredTrainArff() { ArffPropsTableReader
    reader = ArffPropsTableReaderTest.getReader1(); // return the first arff
    having different identifiers as "old test id" // and
    "id the attributes were taken from (train set)"
    // return the first arff id containing "test" in its pathname return
    reader.getIdByArffPath("\_with\_attr\_of\_"); }

---


### getFirstTrainArffFromDb

```
public static int getFirstTrainArffFromDb()
```

---


### getFirstValidArff2FromDb

```
public static int getFirstValidArff2FromDb()
```

---


### getMulan

```
public static MulanArff getMulan(java.lang.String arffPropsPath)
```

---


### getLoadedMulan

```
public static MulanArff getLoadedMulan(java.lang.String arffPropsPath)
```

---


### getWrittenMulan

```
public static MulanArff getWrittenMulan(java.lang.String arffPropsPath)
```

---


### getMulanArff1

```
public static MulanArff getMulanArff1()
```

---


### getMulanArff1LoadedAndWritten

```
public static MulanArff getMulanArff1LoadedAndWritten()
```

---


### getMulanArffTest

```
public static MulanArff getMulanArffTest()
```

---


### getMulanArffTestLoadedAndWritten

```
public static MulanArff getMulanArffTestLoadedAndWritten()
```

---


### getMulanArffTrain

```
public static MulanArff getMulanArffTrain()
```

---


### getMulanArffTrainLoadedAndWritten

```
public static MulanArff getMulanArffTrainLoadedAndWritten()
```


---


|  |  |  |
| --- | --- | --- |
| **Overview** \| **Package** \| **Class** \| **Use** \| **Tree** \| **Deprecated** \| **Index** \| **Help** | |  |
| **PREV CLASS**   **NEXT CLASS** | **FRAMES**    **NO FRAMES**     **All Classes** |  |
| SUMMARY: NESTED \| FIELD \| CONSTR \| METHOD | DETAIL: FIELD \| CONSTR \| METHOD |  |


---
